# CX3CL1-CX3CR1 axis protects retinal ganglion cells by inhibiting microglia activation in a distal optic nerve trauma model

**DOI:** 10.1186/s41232-024-00343-4

**Published:** 2024-06-06

**Authors:** Huan Yu, Bingqiao Shen, Ruiqi Han, Yang Zhang, Shushu Xu, Yumeng Zhang, Yanzhi Guo, Ping Huang, Shouyue Huang, Yisheng Zhong

**Affiliations:** 1 Department of Ophthalmology, Ruijin Hospital Affiliated Medical School, Shanghai Jiaotong University, 197 Ruijin Er Road, Shanghai, 200025 China; 2 Shanghai Key Laboratory for Bone and Joint Diseases, Shanghai Institute of Traumatology and Orthopaedics, Ruijin Hospital Affiliated Medical School, Shanghai Jiaotong University, 197 Ruijin Er Road, Shanghai, 200025 China; 3 Department of Ophthalmology, Shanghai General Hospital (Shanghai First People’s Hospital), Shanghai Jiao Tong University, Shanghai, 200080 China

**Keywords:** CX3CL1-CX3CR1 axis, Microglia, Retinal ganglion cells, Optic nerve trauma

## Abstract

**Background:**

The chemokine CX3CL1 has been reported to play an important role in optic nerve protection, but the underlying mechanism is still unclear. CX3CR1, the only receptor of CX3CL1, is specifically expressed on retinal microglia, whose activation plays a role in the pathological process of optic nerve injury. This study aimed to evaluate whether CX3CL1 exerts optic neuroprotection by affecting the activation of microglia by combining with CX3CR1.

**Methods:**

A mouse model of distal optic nerve trauma (ONT) was used to evaluate the effects of the CX3CL1-CX3CR1 axis on the activation of microglia and survival or axonal regeneration of retinal ganglion cells (RGCs). The activation of microglia, loss of RGCs, and damage to visual function were detected weekly till 4 weeks after modeling. CX3CL1 was injected intravitreally immediately or delayed after injury and the status of microglia and RGCs were examined.

**Results:**

Increases in microglia activation and optic nerve damage were accompanied by a reduced production of the CX3CL1-CX3CR1 axis after the distal ONT modeling. Both immediate and delayed intravitreal injection of CX3CL1 inhibited microglia activation, promoted survival of RGCs, and improved axonal regenerative capacity. Injection with CX3CL1 was no longer effective after 48 h post ONT. The CX3CL1-CX3CR1 axis promotes survival and axonal regeneration, as indicated by GAP43 protein and gene expression, of RGCs by inhibiting the microglial activation after ONT.

**Conclusions:**

The CX3CL1-CX3CR1 axis could promote survival and axonal regeneration of RGCs by inhibiting the microglial activation after optic nerve injury. The CX3CL1-CX3CR1 axis may become a potential target for the treatment of optic nerve injury. Forty-eight hours is the longest time window for effective treatment after injury. The study is expected to provide new ideas for the development of targeted drugs for the repair of optic nerve.

## Background

Optic nerve damage is the main cause of irreversible progressive vision loss, as the axons of retinal ganglion cells (RGCs) that form the optic nerve have limited capacity for regeneration [1, 2]. There are many diseases that cause optic nerve damage, both directly and indirectly, including glaucoma and craniocerebral trauma [3]. Due to the irreversibility of optic nerve injury, the search for optic neuroprotective agents is an important and urgent issue [2].

**Fig. 1:**
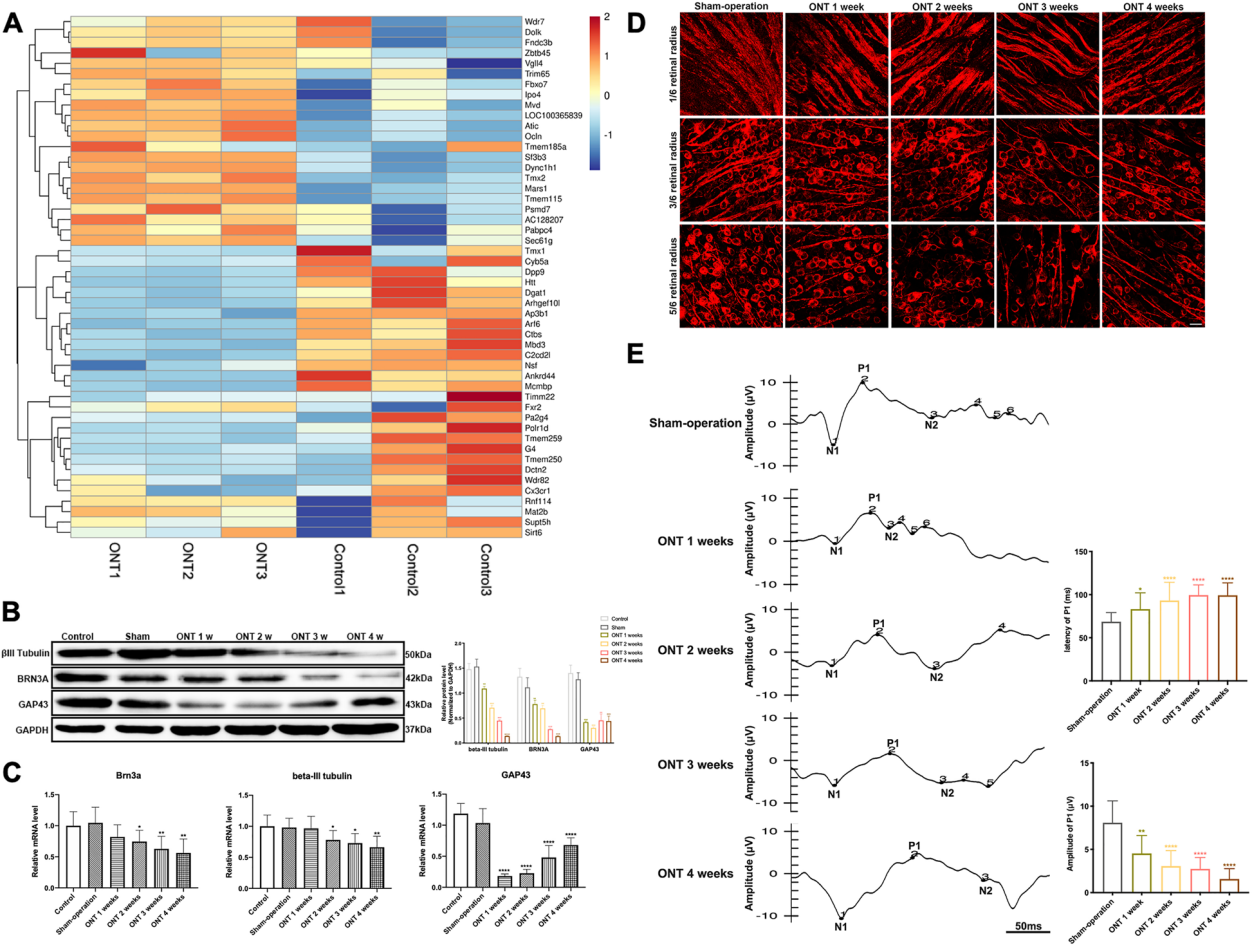
Heat map of DEGs associated with microglia in the mouse retina, changes in βIII-Tubulin, BRN3A, and GAP-43 expressions within mouse retina, and visual function induced by ONT. A, Heat map of DEGs associated with microglia in mouse retina after ONT induction. The expression of gene *CX3CR1* in mouse retina decreased significantly after ONT induction. (− 0.5 < log2FC < 0.5 and *p* < 0.05) (*n* = 3). **B** Protein expressions of βIII-Tubulin, BRN3A, and GAP-43 in mouse retina at 0, 1, 2, 3, and 4 weeks of ONT (*n* = 6). **C** mRNA expressions of βIII-Tubulin, BRN3A, and GAP-43 in mouse retina at 0, 1, 2, 3, and 4 weeks of ONT (*n* = 6). **D** Labeling of βIII-Tubulin in ONT retina. Representative images depict the immunohistochemistry for RGCs and their axons labeled by βIII-Tubulin. Magnification 200, scale bar = 50 μm. **E**, **F** VEP detection waveforms of mice at 0, 1, 2, 3, and 4 weeks of ONT and quantitative analysis of the latency and amplitude of P1 (*n* = 6). Bars indicate the mean, while error bars show the standard deviation. *P < 0.05, **P < .01, ***P < .001, ****P < .0001 versus the control group

In order to explore neuroprotective factors, we constructed a distal optic nerve trauma (ONT) mouse model and extracted RNA from the mouse retina after ONT induction for RNA sequencing. The sequencing results showed that the expression of gene *CX3CR1* in the retina decreased significantly after ONT induction (Fig. 1A). The gene *CX3CR1* encodes protein CX3CR1, and its receptor is CX3CL1.

CX3CL1, also known as Fractalkine (FKN), is a signal chemokine that can be membrane-bound or secreted [4–6], by RGCs in the retina, and is the only member of the CX3C subclass of chemokines [7]. CX3CL1 executes a signal transduction function by combining with its only homologous receptor, CX3CR1, which is specifically expressed by microglia, the primary immune cells in the central nervous system (CNS) or retina [8, 9]. Together, the chemokines and receptors form a signal network between neurons and microglia, known as the CX3CL1-CX3CR1 axis [10].

The neuron-microglia crosstalk enabled by the CX3CL1-CX3CR1 axis has important regulatory functions under both normal physiological and pathological conditions [11]. Microglia are resident immune cells of the CNS and participate in immune regulation [12]. The CX3CL1-CX3CR1 axis is involved in synapse maturation, cell adhesion, microglia migration and activation, and neuron protection [13–15], and can have a protective effect on neurons in CNS neurodegenerative diseases [11, 16]. Under normal physiological conditions, microglia are mostly in a static state, and their synapses are branched to receive signals from the surrounding environment [11]. In pathological processes, microglia can be activated rapidly and are the earliest responding cells in the CNS [17–19]. In neurodegenerative diseases, such as glaucoma, retinitis pigmentosa, and age-related neurodegeneration, retinal microglia produce pro-inflammatory neurotoxic cytokines and eliminate live neurons, including RGCs, through phagocytosis [20–22]. Modulation of the activation state of microglia, along with the consequential chain reactions, within the neuro-immune system can therefore have a significant impact on neuroprotection, as demonstrated in animal models of retinal diseases [23–25]. For example, the dynamic profile of the activation level of microglia can predict the severity of future optic nerve damage, and that inhibiting the pathological activation of microglia could effectively protect RGCs [23, 24]. The specific role of the axis and related mechanisms in optic nerve degeneration is still unclear [2].

Improving the inherently poor axonal regenerative ability of RGCs is potentially key to improving the prognosis of optic nerve injury and neurodegenerative diseases [1, 2]. In adult mammals, RGC axonal regeneration after optic nerve injury is typically limited to the lesion site [1], though regeneration improvements, such as with peripheral nerve graphs and immune modulation, allow axons to grow from the lesion site to the brain [1, 26, 27]. Although the CX3CL1-CX3CR1 axis has been shown to be beneficial to neuroprotection in CNS degeneration [13, 25], the detailed mechanism of the protective effect is still unclear [2]. Similarly, the role of the CX3CL1-CX3CR1 axis in optic neuropathy has not been elucidated, for example, whether it promotes damaged RGC survival and/or stimulates damaged RGCs axonal regeneration. Addressing these knowledge gaps is likely necessary for better facilitating the adult nervous system’s recovery after injury by contributing to a treatment option for optic nerve injury [2].

To determine the potential mechanisms of the CX3CL1-CX3CR1 axis on RGCs survival and axonal regeneration, we used a mouse distal optic nerve injury model to investigate the relationship between CX3CL1-CX3CR1 axis activity indicators, and microglial activation (Ionized calcium binding adaptor molecule-1, Iba-1 and Cluster of differentiation 68, CD68), RGCs survival (βIII-Tubulin and BRN3A) and axonal regeneration (growth associated protein-43, GAP-43) and visual function (F-VEP). The effects of timely and delayed (48 h) CX3CL1 intravitreal injection on the above indicators were tested. Establishing the mechanisms of RGC-microglial network crosstalk after injury may highlight therapeutic targets for RGC survival and axonal regeneration.

## Methods

### Animals

Specific pathogen-free (SPF) standardized male BALA/c mice, aged 4–6 weeks, fed under a 12-h light and dark cycle were used (Zhejiang Vital River Laboratory Animal Technology Co., Ltd., Zhejiang, China). All animal experiment procedures were approved by the institutional review board of Ruijin Hospital, Shanghai, China, and followed the Association for Research in Vision and Ophthalmology (ARVO) guidelines, with effort taken to minimize animal suffering.

## Animal procedures

### Induction of ONT

We developed an optic nerve injury method for mice that avoided non-specific damage to brain tissue and the surrounding elaborate blood vessels [28]. Mice were anesthetized with xylazine (10 mg/kg; Sigma-Aldrich, St. Louis, MO, USA) and ketamine hydrochloride (25 mg/kg; Sigma-Aldrich) by intraperitoneal injection before surgery. The mice were fixed on 3D head stereotaxic apparatus and, after shaving the hair on the top of the head, the skin of the cranial top was cut longitudinally, and the fascia separated to expose the anterior fontanelle. Taking the anterior fontanelle as the basic point, the needle insertion point was determined as 0.5 mm posterior and to the left. A 30-G needle was slowly pierced through the mouse skull to the base, at a depth of approximately 6 mm from the skull surface. Two points of resistance were perceived during the puncture process; (1) when the needle penetrated the optic nerve sheath at approximately 3 mm, causing a slight nystagmus, and (2) at a depth of approximately 6 mm, when the base of the skull was reached. The needle was then withdrawn slowly, the parietal fascia and skin were sutured, and a gatifloxacin ophthalmic ointment (Santen Pharmaceutical, Osaka, Japan) was applied to prevent infection. In mice designated for the sham-operation group, the needle was inserted into the cranial cavity no more than 2 mm.

### Flash visual evoked potentials (F-VEP)

Visual evoked potentials (VEPs) were recorded in mice under ether anesthesia using subdermal needle electrodes (Multi-focal Visual Diagnostic Test System; Model: LKC-UTAS-SBMF; LKC Technologies, Gaithersburg, MD, USA). The collecting electrode, which recorded flash VEP, was placed 3 mm anterior to the tip of the lambdoidal suture to receive the flash stimuli. The reference electrode was placed at the center of the anterior fontanelle and the pin electrode was placed on the tail, acting as the grounding electrode. With the contralateral eye covered by an opaque black eyeshade, flash stimuli from the visual electrophysiological system were presented to one eye, and the VEP responses were recorded in the contralateral visual cortex. The repetition frequency of the flash stimulus was adjusted to 2.0 Hz, the light intensity of the flash stimulus was set at 0 dB (3.0 cd·s·m − 2) and the response duration was set at 300 ms. An average of 80 responses was performed and the latency and amplitude of the P1 wave were observed, and each eye was measured 3 consecutive times to obtain an average value.

### Intravitreal injection

Intravitreal injection was conducted as previously described [29], with minor modifications. The pupil of the anesthetized eye was dilated with tropicamide drops. A 32-gauge needle was then inserted 1 mm behind the temporal limbus and directed toward the optic nerve head to make an entrance for injection. Subsequently, 1 μL of the designated liquid was injected into the vitreous cavity using a microinjector (Hamilton Bonaduz AG, Switzerland) under a stereoscopic microscope (Carl Zeiss Microscopy, Jena, Germany).

### Intraperitoneal injection of PLX3397

PLX3397 intraperitoneal injection was conducted as previously described [30]. Briefly, mice underwent intraperitoneal injections of PLX3397 (MedChemExpress, HY-16749) at a dosage of 1 mg per kilogram body weight per day. PLX3397 was formulated in a solution comprising 1% dimethyl sulfoxide (DMSO), 45% polyethylene glycol 300 (PEG-300), 5% Tween 80, and 49% saline. Control mice received intraperitoneal injections of the solvent vehicle using an identical regimen.

### Immunofluorescence

The eyeballs or cells adhered to coverslips were fixed in paraformaldehyde for 1 h, and the retinal neuroepithelial layers were separated to acquire retina stretched preparation. To prepare retinal tissue sections, the ocular anterior segments were removed to retain eyecups, which were frozen in an Optimum Cutting Temperature compound (OCT; Sakura Finetek, Torrance, CA, USA), and 10-μm-thick cryosections were cut and air-dried. The retinal slices or sections of the cells on coverslips were permeated with cold 0.25% Triton X-100 solution (Sigma-Aldrich) for 30 min, then blocked in 1% BSA at room temperature for 1 h. Samples were incubated with rabbit-derived polyclonal anti-CX3CL1 (1:1000 dilution, ab25088; Abcam, Cambridge, UK), monoclonal anti-BRN3A (1:100 dilution, ab245230; Abcam), monoclonal anti-βIII Tubulin (1:500 dilution, ab52623; Abcam), monoclonal anti-GAP43 (1:100 dilution, ab75810; Abcam), or monoclonal anti-Iba-1 (1:500 dilution, ab178846; Abcam) primary antibodies at 4 °C overnight using single or combined application as needed. Samples were then incubated with Texas-red conjugated donkey anti-rabbit (1:1000 dilution; Abcam) at room temperature for 1 h. The samples were further incubated with 4′,6-diamidino-2-phenylindole (DAPI; Sigma-Aldrich) for 5 min and examined under microscopy (Carl Zeiss Microscopy).

## Experimental designs

### Temporal dynamics of RGC-microglial network crosstalk post-injury

A total of 24 mice were subjected to ONT (treatment group), 6 mice underwent the sham ONT operation and 6 were designated to the control group, with no ONT or sham ONT. At 0 weeks post-ONT, 6 mice from the treatment group and the sham + operation group underwent F-VEP and were subsequently sacrificed, in addition to mice from the control group (*n* = 6) for gene and protein expression and immunohistochemistry. Six mice from the sham + operation group underwent F-VEP and were subsequently sacrificed at 0 weeks post-ONT, while 6 mice from the treatment group underwent F-VEP and were sacrificed at each of 0, 1-, 2-, 3-, and 4 weeks post-ONT. Mice in the control group were sacrificed at week 0. Samples were then taken for protein and gene expression and immunofluorescent histochemistry.

### Inhibition of the CX3CL1-CX3CR1 axis

A total of 12 mice were subjected to ONT and a further 12 underwent the sham operation. Mice then underwent intravitreal injection with either 1 μl of CX3CR1 neutralizing antibody (Anti-CX3CR1, 5 μg/mL, diluted in PBS (phosphate buffered saline); GTX27200, Genetex) (*n* = 6 per treatment group) or PBS (control, *n* = 6 per treatment group). Mice from all treatment groups (sham-operated + PBS, sham-operated + Anti-CX3CR1, ONT 1 week + PBS, and ONT 1 week + Anti-CX3CR1) were sacrificed for western blot analysis and immunohistochemistry at 1 week post-ONT.

### Screening for optimal intravitreal injection of CX3CL1

A total of 72 mice underwent ONT and were then divided equally into ONT + CX3CL1 and ONT + PBS treatment groups. In the ONT + CX3CL1 groups, six mice were then given an intravitreal injection of each of the CX3CL1 concentrations (1 ng/mL, 5 ng/mL, 10 ng/mL, 15 ng/mL, 20 ng/mL, diluted in PBS; Merck KGaA, Darmstadt, Germany) and mice from the ONT + PBS were injected with PBS. To test the optimal time to administer CX3CL1, a further 48 mice underwent ONT and were divided equally into ONT + CX3CL1 and ONT + PBS treatment groups. An intravitreal injection of either CX3CL1 (5 ng/mL) or PBS (depending on the treatment group) was given immediately or 24, 48, and 72 h after ONT induction. In a final experiment, 12 mice underwent ONT and underwent intravitreal injection of CX3CL1 (5 ng/ml) or PBS at 48 h post-ONT. For all experiments, mice were sacrificed at 1-week post-ONT to examine the effect of CX3CL1 on retinal microglia and RGCs.

## Parameters

### Western blot analysis

Collected retinas or cells were immersed, on ice, in RIPA lysis buffer (Sigma-Aldrich) with a Complete Protease Inhibitor cocktail (Roche Applied Science, Penzberg, Germany). Lysates were homogenized and centrifuged. The supernatant was collected and protein concentrations were quantified. Protein samples of equal amounts were separated on 12.5% SDS-PAGE and then electrotransferred onto a polyvinylidene fluoride membrane (PVDF; Merck Millipore, Billerica, MA). After blocking for 10 min in rapid blocking liquid, membranes were incubated with primary antibodies at 4 ℃ overnight, and then incubated with secondary antibodies (Abcam) conjugated to horseradish peroxidase (HRP) for 1 h at room temperature. The protein expression level was determined using densitometric analysis and normalized to GAPDH expression. The primary antibodies were: Anti-GAPDH (1:10,000 dilution; Abcam, ab8245), Anti-CX3CL1 (1:1000 dilution; Abcam, ab25088), Anti-CX3CR1 (1:1000 dilution; Abcam, ab8020), Anti-BRN3A (1:1000 dilution; Abcam, ab245230), Anti-βIII Tubulin (1:500 dilution; Abcam, b52623), Anti-GAP43 (1:1000 dilution; Abcam, ab75810), Anti-Iba-1 (1:1000 dilution; Abcam, ab178846), Anti-CD68 (1:1000 dilution; Abcam, ab125212).

### Quantitative real-time-PCR

Total RNA was isolated from mouse retina or microglia cells using Trizol reagent (Invitrogen, Carlsbad, CA) following the manufacturer’s instructions. The RNA was converted to cDNA using the reverse transcriptase kit PrimeScript RT Master Mix (Takara Bio, Inc., Shiga, Japan). Primers were designed using Primer Premier 5.0 software (Thermo Fisher Scientific, Grand Island, NY, USA), and primer pairs used were:

- β-actin
- (F: CACTATCGGCAATGAGCGGTTCC, R: CAGCACTGTGTTGGCATAGAGGTC),
- β3-Tubulin
- (F: AAGAACATGATGGCTGCCTGTGAC, R: TCTGCTCGTCCACCTCCTTCATAG),
- Brn3a
- (F: ACGCTCTCGCACAACAACATGATC, R: GCTCCGGCTTGTTCATTTTCTCAC),
- GAP43
- (F: GCTGTGCTGTATGAGAAGAACCAAAC, R: GGTCGCAGCCTTATGAGCCTTATC),
- CD68
- (F: CCTCTTGCTGCCTCTCATCATTGG, R: GGCTGGTAGGTTGATTGTCGTCTG),
- Iba-1
- (F: AATGATGAGGATCTGCCGTCCAAAC, R: TCTCTTCAGCTCTAGGTGGGTCTTG)

Quantitative real-time PCR was performed using the SYBR Premix Ex Taq II kit (Takara Bio, Inc.) in a total volume of 20 μL on a 7500 real-time PCR (Applied Biosystems, Foster City, CA, USA), employing a procedure as 95 ℃ for 30 s, 40 cycles of 95 ℃ for 5 s, 60 ℃ for 34 s, and 72 ℃ for 30 s. mRNA contents of each sample were detected in duplicates. β-Actin was used as a reference gene. Relative quantification of each gene expression was calculated using the 2^−ΔΔCt^ method as described previously [31].

### Statistical analysis

All data are presented as mean ± standard deviation. Statistical differences between groups were estimated using a Student’s *t*-test or one-way ANOVA, and *P* < 0.05 was considered to be statistically significant. Statistical analyses were performed with GraphPad Prism 7.0.

## Results

### Temporal dynamics of RGC-microglial network crosstalk post-injury

The protein expressions of βIII-Tubulin (*p* = 0.0056, 0.0005, 0.001, < 0.0001), BRN3A (*p* = 0.0056, 0.0005, 0.001, < 0.0001) and GAP-43 (*p* = 0.0005, 0.0003, 0.0014, 0.0009) decreased significantly after ONT compared to those of the control group (Fig. 1B). While βIII-Tubulin and BRN3A protein expression decreased week-on-week from week 1, GAP43 expression decreased at weeks 1 and 2 post-ONT then increased slightly in both weeks 3 and 4 (Fig. 1B). The mRNA expressions of βIII-Tubulin, BRN3A and GAP-43 followed similar patterns to the protein expression, with βIII-Tubulin (*p* = 0.7240, 0.0131, 0.0155, 0.0034) and BRN3A (*p* = 0.0895, 0.0381, 0.0041, 0.0027) decreasing gradually from week 2 to 4 post-ONT while GAP43 (*p* < 0.0001, < 0.0001, < 0.0001, < 0.0001) showed a large initial decrease and then gradually increased in weeks 2 to 4 (Fig. 1C). Immunofluorescence for βIII-Tubulin in retinas post-ONT showed RGC number gradually decreased with time post-ONT, accompanied with reduced number of axon bundles, as compared to the control group (Fig. 1D). The P1 amplitude wave (*p* = 0.0001, < 0.0001, < 0.0001, < 0.0001) of flash visual evoked potentials (F-VEP) gradually decreased with time post-ONT and the latency (*p* = 0.0022, < 0.0001, < 0.0001, < 0.0001) gradually prolonged (Fig. 1E), indicating RGCs loss and visual function impairment.

**Fig. 2:**
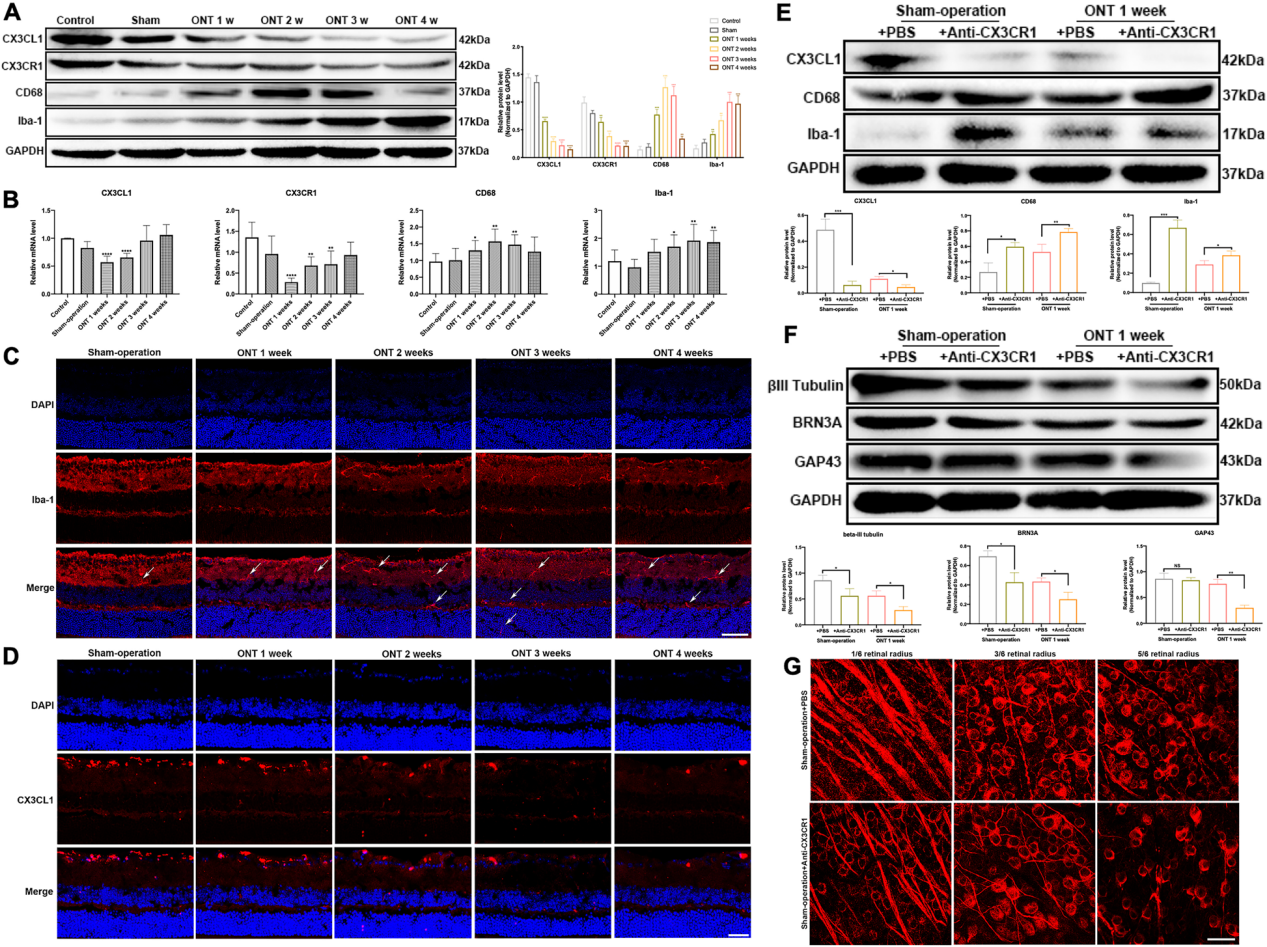
Changes of Iba-1, CD68, CX3CL1, and CX3CR1 expressions within mouse retina induced by ONT and the effects of Anti-CX3CR1 injected intravitreally on retinal microglia and RGCs. **A** Protein expressions of Iba-1, CD68, CX3CL1, and CX3CR1 in mouse retina at 0, 1, 2, 3, and 4 weeks of ONT (*n* = 6); B, mRNA expressions of Iba-1, CD68, CX3CL1, and CX3CR1 in mouse retina at 0, 1, 2, 3, and 4 weeks of ONT (*n* = 6). **C** Labeling of microglia in ONT retina. Representative images depict the immunohistochemistry for microglia labeled by Iba-1, with white arrows pointing to microglia **D** Labeling of CX3CL1 in the ONT retina. Representative images depict the immunohistochemistry for CX3CL1 distributed in the retinal ganglion cell layer (GCL). E, Western blot analysis and densitometry quantification of CX3CL1, CD68, and Iba-1 expressions in the sham-operated + PBS group, sham-operated + Anti-CX3CR1 group, ONT 1 week + PBS group, and ONT 1 week + Anti-CX3CR1 group (*n* = 6).** F** Western blot analysis and densitometry quantification of βIII-Tubulin, BRN3A, and GAP-43 expressions in the sham-operated + PBS group, sham-operated + Anti-CX3CR1 group, ONT 1 week + PBS group, and ONT 1 week + Anti-CX3CR1 group (*n* = 6). **G** Labeling of βIII-Tubulin in ONT retina of in sham-operated + PBS group, sham-operated + Anti-CX3CR1 group (*n* = 6). Representative images depict the immunohistochemistry for RGCs and their axons labeled by βIII-Tubulin. Magnification 200, scale bar = 50 μm; Bars indicate mean, while error bars show standard deviation. *P < 0.05, **P < .01, ***P < .001, ****P < .0001 versus the control group

The protein expression of CX3CL1 (*p* < 0.0001, < 0.0001, < 0.0001, < 0.0001) and CX3CR1 (*p* = 0.0091, 0.0008, < 0.0001, < 0.0001) decreased after ONT compared to the control groups, while expression of the microglia markers Iba-1 (*p* = 0.0026, 0.0011, 0.0007, 0.0008) and CD68 (*p* = 0.0005, 0.0004, 0.0005, 0.0083) of mouse retina increased (Fig. 2A). The mRNA expression trends of CX3CL1 (*p* < 0.0001, < 0.0001, 0.8054, 0.5978) and CX3CR1 (*p* < 0.0001, 0.0084, 0.0042, 0.0618) and the microglia markers were broadly consistent with the protein expression (Fig. 2B). Immunofluorescent-labelled Iba-1 highlighted retinal microglia, which increased in number and migrated towards the outer nuclear layer while synapses gradually shortened after ONT (Fig. 2C). Labeling of CX3CL1 in ONT retinas showed distribution in the RGC layer, with a gradual decrease in secretion with time after ONT induction (Fig. 2D).

### Inhibition of the CX3CL1-CX3CR1 axis

Western blot results show a significant decrease in CX3CL1 (*p* = 0.0011, 0.0107) expression with intravitreal injection of Anti-CX3CR1, compared to PBS injection groups, and an increase in expressions of Iba-1 (*p* = 0.0003, 0.0488) and CD68 (*p* = 0.0123, 0.0148) (Fig. 2E). The protein expression of BRN3A (*p* = 0.0154, 0.0167) and βIII-Tubulin (*p* = 0.0366, 0.0137) significantly decreased with intravitreal injection of Anti-CX3CR1, compared to the control, and the expression of GAP43 (*p* = 0.7177, 0.0013) was significantly reduced in the ONT 1 week + Anti-CX3CR1 group compared to controls (Fig. 2F). Immunofluorescence analysis showed that the appeared of RGCs and axons stained positive with βIII-Tubulin reduced after Anti-CX3CR1 injection (Fig. 2G).

### Screening for optimal intravitreal injection of CX3CL1

**Fig. 3:**
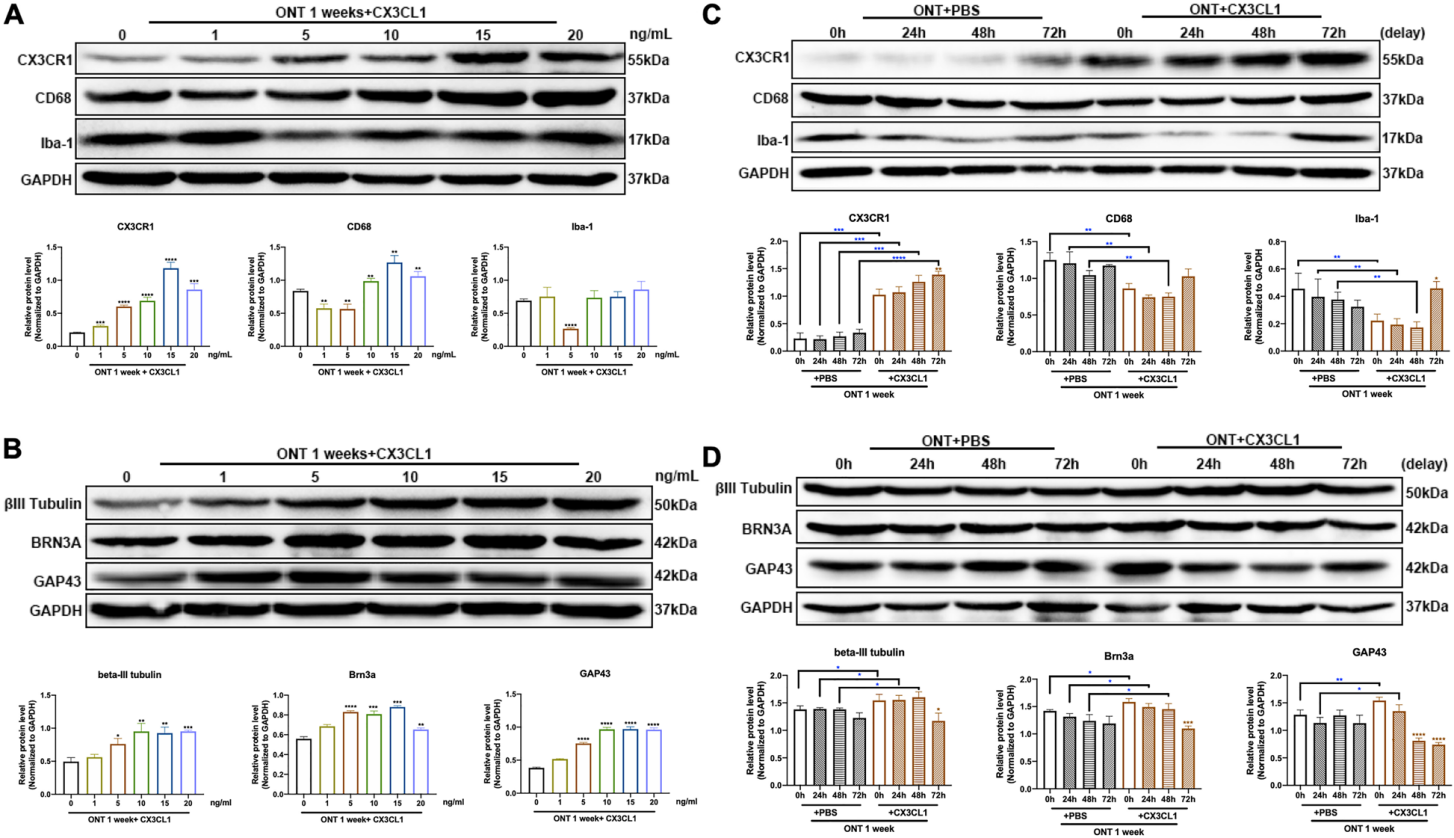
The effects of CX3CL1 injected intravitreally on retinal microglia and RGCs. **A** Western blot analysis and densitometry quantification of CX3CR1, CD68, and Iba-1 expressions within mouse retina in PBS and different concentrations of CX3CL1 injected groups (*n* = 6). **B** Western blot analysis and densitometry quantification of βIII-Tubulin, BRN3A, and GAP-43 expressions within mouse retina in PBS and different concentrations of CX3CL1 injection groups (*n* = 6). **C** Western blot analysis and densitometry quantification of CX3CR1, CD68, and Iba-1 expressions within mouse retina in groups of PBS or CX3CL1 injection at different time points (*n* = 6). **D** Western blot analysis and densitometry quantification of βIII-Tubulin, BRN3A, and GAP-43 expressions within mouse retina in groups of PBS or CX3CL1 injection at different time points (*n* = 6). Bars indicate the mean, while error bars show the standard deviation. *P < 0.05, **P < 0.01, ***P < 0.001, ****P < 0.0001 versus the PBS injected group

The expression of CX3CR1 (*p* = 0.0003, < 0.0001, < 0.0001, < 0.0001, 0.0002) increased in parallel with the increment of the concentration of CX3CL1 injected, reaching a peak at 15 ng/mL and remaining stable thereafter (Fig. 3A). Microglia marker Iba-1 (*p* = 0.4937, < 0.0001, 0.5296, 0.2651, 0.0784) and CD68 (*p* = 0.0030, 0.0039, 0.0062, 0.0024, 0.0072) expression decreased to a minimum at 5 ng/mL and increasing thereafter (Fig. 3A). The expression of RGCs and axon markers, BRN3A (*p* = 0.0987, < 0.0001, 0.0003, 0.0007, 0.0033), βIII-Tubulin (*p* = 0.1888, 0.0102, 0.0043, 0.0023, 0.0002), and GAP43 (*p* < 0.0001, < 0.0001, < 0.0001, < 0.0001, < 0.0001) increased with the concentrations gradient of CX3CL1 up to 5 ng/mL and remained stable or slightly decreased for higher concentrations (Fig. 3B).

**Fig. 4:**
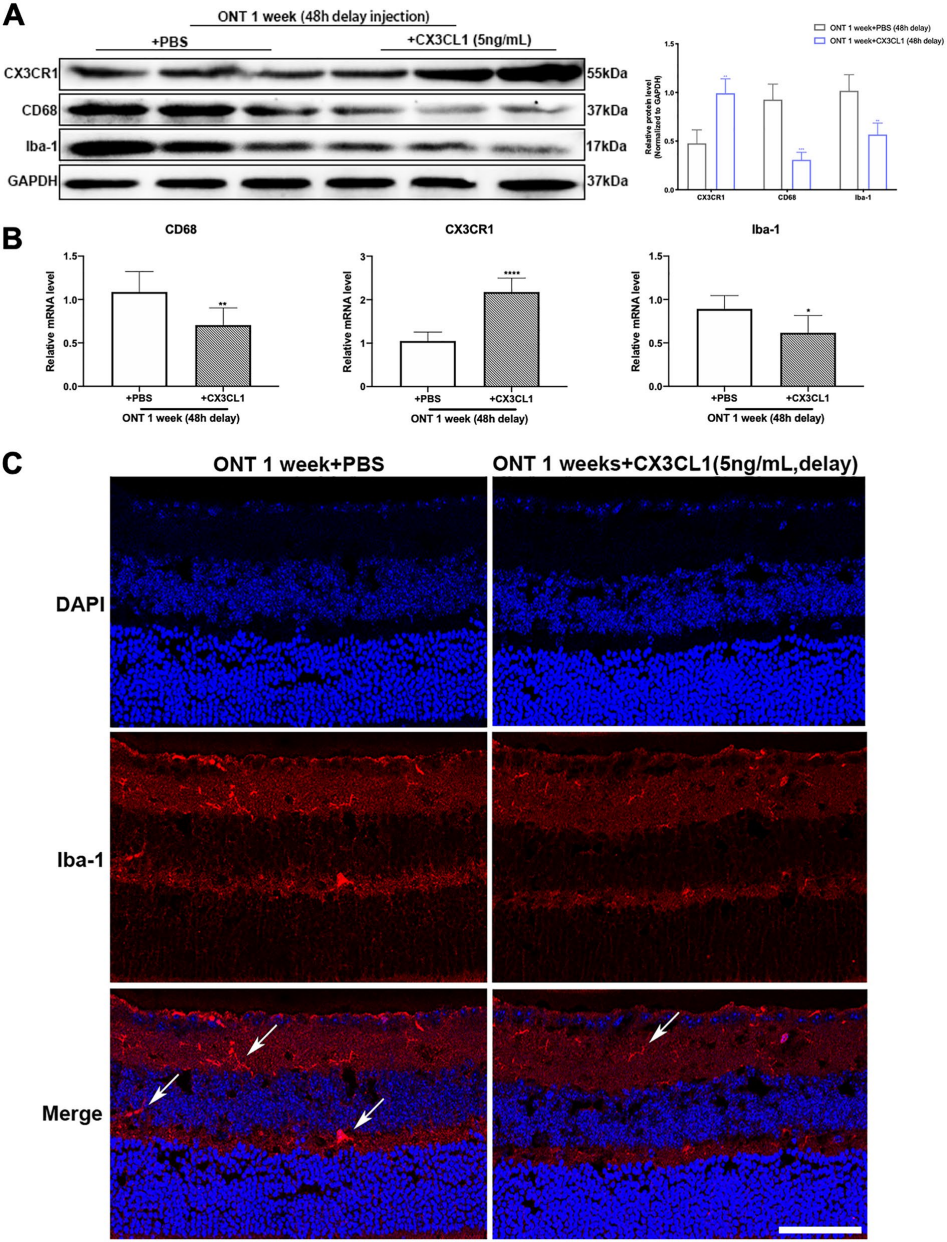
The effects of 48 h delayed intravitreal injection of CX3CL1 on expression of CX3CR1, CD68, and Iba-1. **A** Western blot analysis and densitometry quantification of CX3CR1, CD68, and Iba-1 expressions within mouse retina in PBS and CX3CL1 delayed injection groups (*n* = 6). **B** mRNA expressions of CX3CR1, CD68 and Iba-1 within mouse retina in PBS and CX3CL1 delayed injection groups (*n* = 6). **C** Immunofluorescence staining for Iba-1 in PBS and CX3CL1 delayed injection groups. Representative images show the immunohistochemistry for Iba-1 (red), and DAPI (blue), with white arrows pointing to microglia, magnification 200, scale bar = 50 μm. Bars indicate the mean, while error bars show the standard deviation. *P < 0.05, **P < 0.01, ***P < 0.001, ****P < 0.0001 versus the PBS injected group

**Fig. 5:**
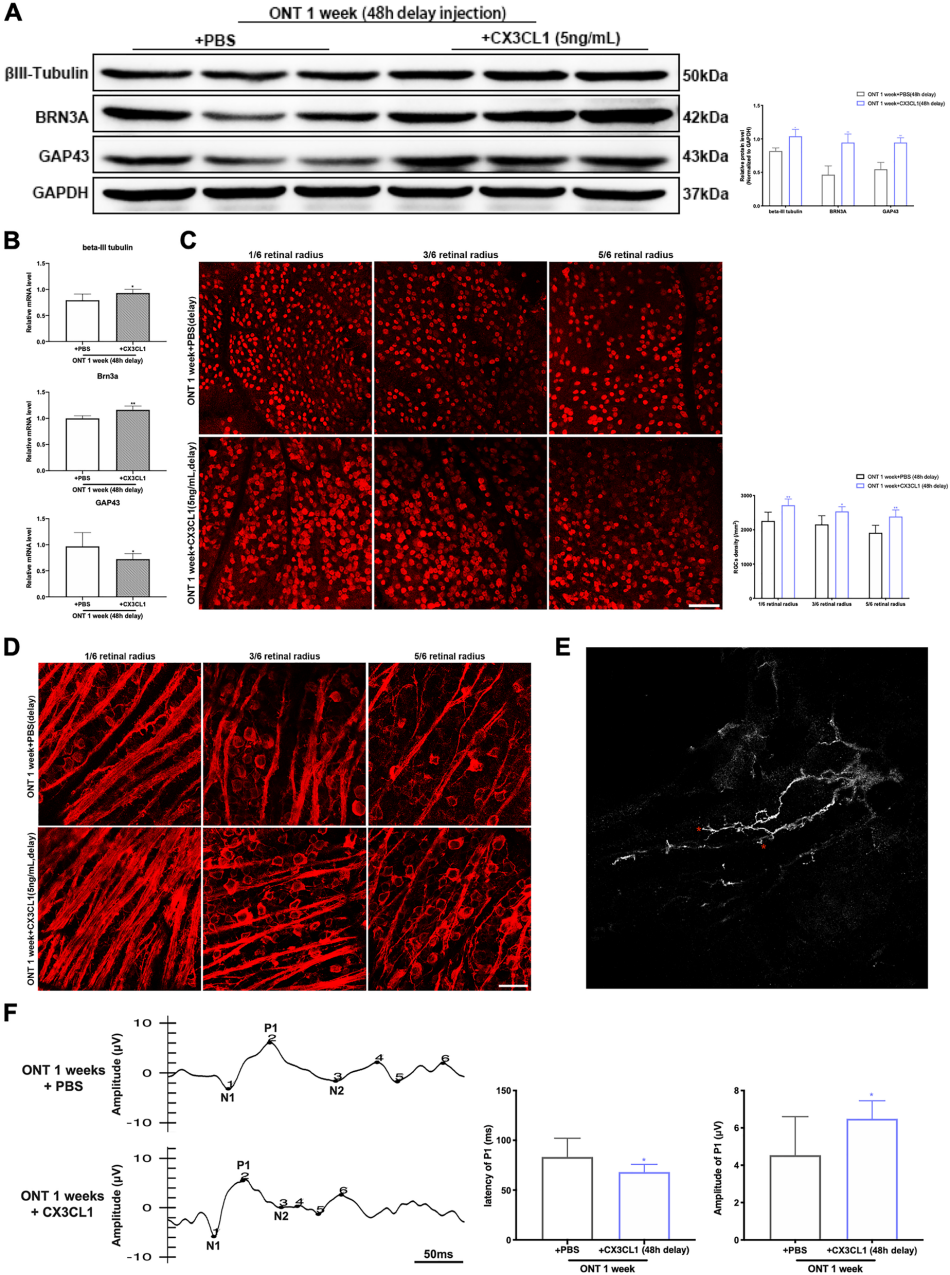
The effects of 48 h delayed intravitreal injection of CX3CL1 on expressions of βIII-Tubulin, BRN3A, and GAP-43. **A** Western blot analysis and densitometry quantification of βIII-Tubulin, BRN3A, and GAP-43 expressions within mouse retina in PBS and CX3CL1 delayed injection groups (*n* = 6). **B** mRNA expressions of βIII-Tubulin, BRN3A, and GAP-43 within mouse retina in PBS and CX3CL1 delayed injection groups (*n* = 6). **C** Immunofluorescence staining and quantitative analysis for RGCs in PBS and CX3CL1 delayed injection groups. Representative images show the immunohistochemistry for RGCs labeled by BRN3A. **D** Immunofluorescence staining for βIII-Tubulin in PBS and CX3CL1 delayed injection groups. Representative images show the immunohistochemistry for RGCs and axons labeled by βIII-Tubulin; magnification 200, scale bar = 50 μm. **E** Immunofluorescence staining for GAP43 in CX3CL1 delayed injection group. A swelling (red asterisk) was seen at the tip of the regenerating buds of RGC in ONT mice after intravitreal injection of CX3CL1. **F** F-VEP detection waveforms of ONT mice in PBS or CX3CL1 injection groups and quantitative analysis of the latency and amplitude of P1 (*n* = 6). Bars indicate the mean, while error bars show the standard deviation. *P < 0.05, **P < 0.01, ***P < 0.001, ****P < 0.0001 versus the PBS injected group

The expression of CX3CR1 (*p* = 0.0006, 0.0002, 0.0003, < 0.0001) significantly increased after CX3CL1 injection at different timepoints, while the expressions of Iba-1 (*p* = 0.0076, 0.0098, 0.0068, 0.1066) and CD68 (*p* = 0.0053, 0.0070, 0.0033, 0.0647) decreased, compared with the control (PBS) injection group (Fig. 3C). Conversely, the expression of BRN3A (*p* = 0.0123, 0.0226, 0.0215, 0.3319) and βIII-Tubulin (*p* = 0.0299, 0.0333, 0.0189, 0.6215) and GAP43 (*p* = 0.0078, 0.0094, 0.0908, 0.1318) marked increased (Fig. 3D). There were statistical differences in the 24 h and 48 h CX3CL1 injection groups, compared with their respective control groups. There were no significant differences in marker expression with CX3CL1 injection at 72 h after ONT as compared with the PBS injection group (Fig. 3C, D). The expression of CX3CR1 (*p* = 0.0050) was significantly increased, and that of Iba-1 (*p* = 0.0091) and CD68 (*p* = 0.0010) were decreased, after CX3CL1 injection at 48 h post-ONT, as compared with control (PBS) injection groups (Fig. 4A, B). Immunofluorescence labelling of Iba-1 in the retina shows a decrease in microglia after CX3CL1 injection, along with cell body shrinkage and attenuation of migration ability towards the outer nuclear layer (Fig. 4C). Injection of 5 ng/mL of CX3CL1 into the vitreous cavity at 48 h after ONT showed an increase in BRN3A (*p* = 0.0041), βIII-Tubulin (*p* = 0.0274) and GAP43 (*p* = 0.0050) expression as compared to controls (Fig. 5A, B). The density of BRN3A-labeled RGCs and βIII-Tubulin-labeled axons increased after CX3CL1 injection (Fig. 5C, D). A swelling was observed at GAP43-labeled synaptic terminals after CX3CL1 injection (Fig. 5E) and F-VEP testing showed that the latency (*p* = 0.0272) of P1 was shortened and amplitude (*p* = 0.0111) increased after CX3CL1 injection, compared to the control (ONT group with injected PBS (Fig. 5F).

## Discussion

Optic nerve trauma (ONT), with resultant vision loss, is largely considered irreversible due to the limited regenerative capacity of retinal ganglion cell (RGC) axons [2]. Modulation of CNS immune cells, microglia, in the retina can be both neuroprotective and neuropathological [25, 32–34], suggesting RGC-microglial signaling network modulation could promote RGC regeneration [1, 2, 35]. We document the temporal dynamics of RGC-microglial network crosstalk post-ONT and demonstrate that chemokine-receptor (CX3CL1-CX3CR1) axis inhibition reduces RGC count and activates microglia. Furthermore, injection of CX3CL1 reduced microglia activity and promoted RGC survival and regeneration and could therefore be explored further as a potential therapeutic option for ONT.

### Temporal dynamics of RGC-microglial network crosstalk post-injury

The controlled induction of ONT using a 30-G needle inserted 6 mm into the mouse skull was effective, presenting a different model method e.g., [36, 37], and led to significant RGC loss, indicated by RGCs markers (βIII-Tubulin and BRN3A) and visual function impairment. These findings are consistent with RGC death associated with many eye diseases [38], potentially due to increased oxidative stress, apoptosis [39, 40], and phagocytosis by microglia [20–22], as well as traumatic optic neuropathy (TON) as a result of injury [41]. The axonal growth-associated protein (GAP-43) is expressed during axonal and synaptic growth [42], and expression in mouse retinas initially declined post-ONT and then gradually increased towards control levels. Injury-induced GAP-43 expression has previously been reported [43], the appearance of GAP-43 immunoreactivity is also considered a sign of RGC axon regeneration or sprouting [44]. The overall suppression in our study suggests a potential inhibition of axonal growth. ONT induction also caused an increase in CD68 and Iba-1 expression in mouse retina, the former of which is related to immune cell regulation while the latter is specific to microglia activation [45, 46]. This is consistent with neuronal injury initiating the process of early activation of microglia, phagocytosis of RGCs [20–22]) and direct communication between neurons and microglia [12], such as via the CX3CL1-CX3CR1 axis. In our study, CX3CL1-CX3CR1 axis activity was suppressed after ONT, potentially consistent with axis signal disruption playing a key role in CNS pathogenesis [11], enabling uncontrolled microglial activation with damaging neurotoxicity [25, 32] and RGC loss via phagocytosis [47].

### Inhibition of the CX3CL1-CX3CR1 axis

Inhibition of the CX3CL1-CX3CR1 axis led to differential modulation of microglia and RGCs upon ONT, highlighting its involvement in microglia regulation, RGCs survival, and nervous system homeostasis. Inhibition of the CX3CL1-CX3CR1 axis using a CX3CR1 neutralizing antibody (Anti-CX3CR1) led to microglia activation, fewer RGCs and suppressed axonal growth and neurogenesis, as indicated by reduced βIII-Tubulin [48] and GAP-43 expression [42]. Findings were consistent with Wang et al. (2014) [47], who found that deletion of CX3CR1 enhanced the neurotoxicity of activated microglia in the mouse glaucoma model, which in turn caused more extensive loss of RGCs. Conversely, a high level of endogenous CX3CL1 expressed by neurons maintains microglia in a relatively quiescent state [49, 50]. These findings suggest that increasing the activity of the CX3CL1-CX3CR1 axis, may have a protective effect on RGB [23, 24] in the ONT scenario, and limit excessive activation of microglia that leads to harmful neurotoxity [33, 34].

### Screening for optimal intravitreal injection of CX3CL1

Intravitreal injection of CX3CL1 at 5 ng/mL optimally activated the CX3CL1-CX3CR1 axis and appeared to moderate microglia activity, with suppression of CD68 and Iba-1 [45, 46] when administered at 48 h post-ONT. The upregulated expression of βIII-Tubulin, BRN3A, and GPA43 post-ONT with an injection of CX3CL1 as compared to controls, suggests a limiting effect on microglia activity [49, 50] and effective protection of RGC [23, 24]. Potential neuroprotective effects of CX3CL1-limited microglia activity [33, 34] likely enabled the axonal regeneration observed post-injury as swelling on GAP43-labeled synaptic terminals of RGCs. While the limited regenerative ability of RGCs has been extensively explored [2], axonal regeneration in the CNS can be promoted [51] by various chemokines, cytokines, and tumor suppressors [52, 53]. For example, injection of zymosan into the eyes [54, 55] and Methylene blue in the optic nerve transection model [56] contribute to neuronal regeneration capacity. Similar to the regeneration observed in our study, Fung et al. (2020) [56] reported the phenomenon of axonal sprouting from damaged RGCs. Our findings therefore suggest that modulation of the CX3CL1-CX3CR1 axis via intravitreal injection of CX3CL1 could help restore ONT-induced RGCs damage and promote RGCs survival and axonal regeneration.

### Microglia inhibition and neuroprotection

**Fig. 6:**
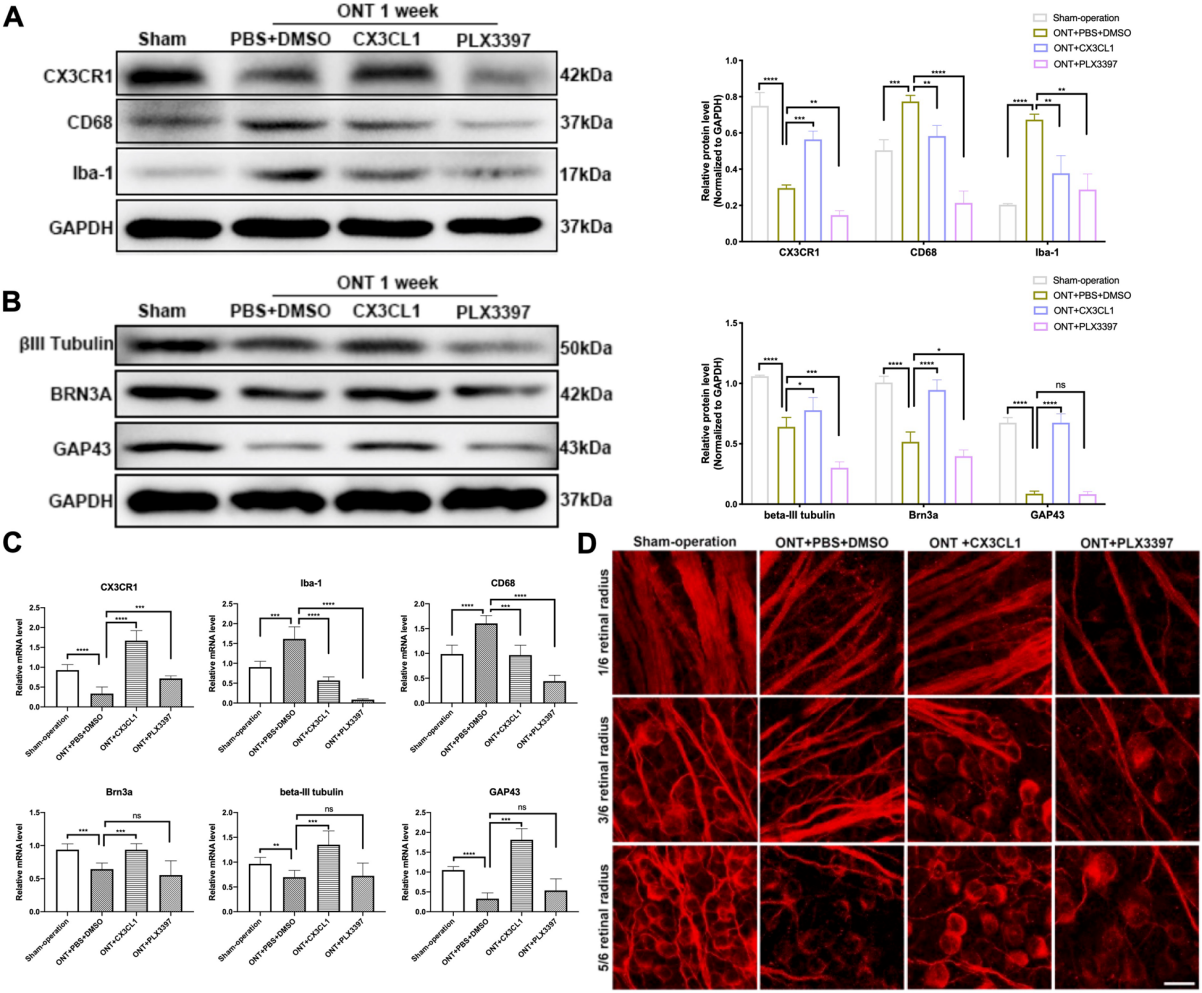
The effects of CX3CL1 and PLX3397 intervention on retinal microglia and RGCs. **A** Western blot analysis and densitometry quantification of CX3CR1, CD68, and Iba-1 expressions within mouse retina in sham-operation, ONT + PBS + DMSO, ONT + CX3CL1 injection, ONT + PLX3397 injection groups (*n* = 6). **B** Western blot analysis and densitometry quantification of βIII-Tubulin, BRN3A, and GAP-43 expressions within mouse retina in sham-operation, ONT + PBS + DMSO, ONT + CX3CL1 injection, ONT + PLX3397 injection groups (*n* = 6). **C** mRNA expressions of CX3CR1, CD68, Iba-1, βIII-Tubulin, BRN3A, and GAP-43 within mouse retina in sham-operation, ONT + PBS + DMSO, ONT + CX3CL1 injection, ONT + PLX3397 injection groups (*n* = 6). **D** Labeling of βIII-Tubulin in mouse retina of sham-operation, ONT + PBS + DMSO, ONT + CX3CL1 injection, ONT + PLX3397 injection groups. Representative images depict the immunohistochemistry for RGCs and their axons labeled by βIII-Tubulin. Magnification 400, scale bar = 50 μm. Bars indicate the mean, while error bars show the standard deviation. *P < 0.05, **P < 0.01, ***P < 0.001, ****P < 0.0001

Numerous inhibitors of microglia, such as PLX3397, exist within the realm of neuroprotection research [57, 58]. One may ponder whether these inhibitors could emulate the neuroprotective effects observed through the inhibition of microglial activation, as is the case with the CX3CL1-CX3CR1 axis. To explore this intriguing query, we elected to employ PLX3397 in a preliminary controlled experiment. PLX3397, a small-molecule inhibitor of colony-stimulating factor 1 receptor (CSF-1R) [59], is known for its potent microglial depletion capabilities, particularly within the retinal environment [30, 60]. In this study, we intervened in ONT-induced mice with either CX3CL1 or PLX3397 and compared the respective effects. We assessed the activation state of microglia, alongside the survival and regenerative capacities of RGCs. Our findings indicated that while both CX3CL1-CX3CR1 activation and PLX3397 intervention suppress the expression of Iba-1 and CD68 (Fig. 6A, C), a marked disparity in the impact on RGCs was observed. Contrary to CX3CL1-CX3CR1, PLX3397 did not enhance the survival and regeneration of RGCs post-ONT; in fact, it significantly reduced the expression levels of βIII-Tubulin, Brn3a, and GAP-43 (Fig. 6B, C). Additionally, retinal staining for βIII-Tubulin revealed that RGC and axon bundle counts significantly increased following CX3CL1-CX3CR1 activation but markedly decreased in the PLX3397-treated group (Fig. 6D), suggesting potential neurotoxic effects in the latter. Previous literature corroborated the microglial depletion effect of PLX3397 and its potential to exacerbate neuroinflammation and cerebral damage during ischemic events [61, 62], which may elucidate the outcomes observed in our experiment. The dual role of microglia in the nervous system and retina—as both protective and potentially harmful—has been extensively documented. This underscores the critical need for precise modulation in strategies aiming at neuroprotection. Thus, our results contribute to the understanding that while microglial inhibition can be beneficial under certain circumstances, indiscriminate inhibition may lead to adverse outcomes, highlighting the complexity of microglial dynamics in neurodegenerative disease contexts.

### Study limitations

Since there is no commonly acknowledged in vitro model that simulates optic nerve truncation injury currently, the lack of in vitro experiments turns out to be a limitation in this study. Besides, expanding the sample size and further looking for evidence of RGC regeneration will contribute to our conclusion.

## Conclusion

**Fig. 7:**
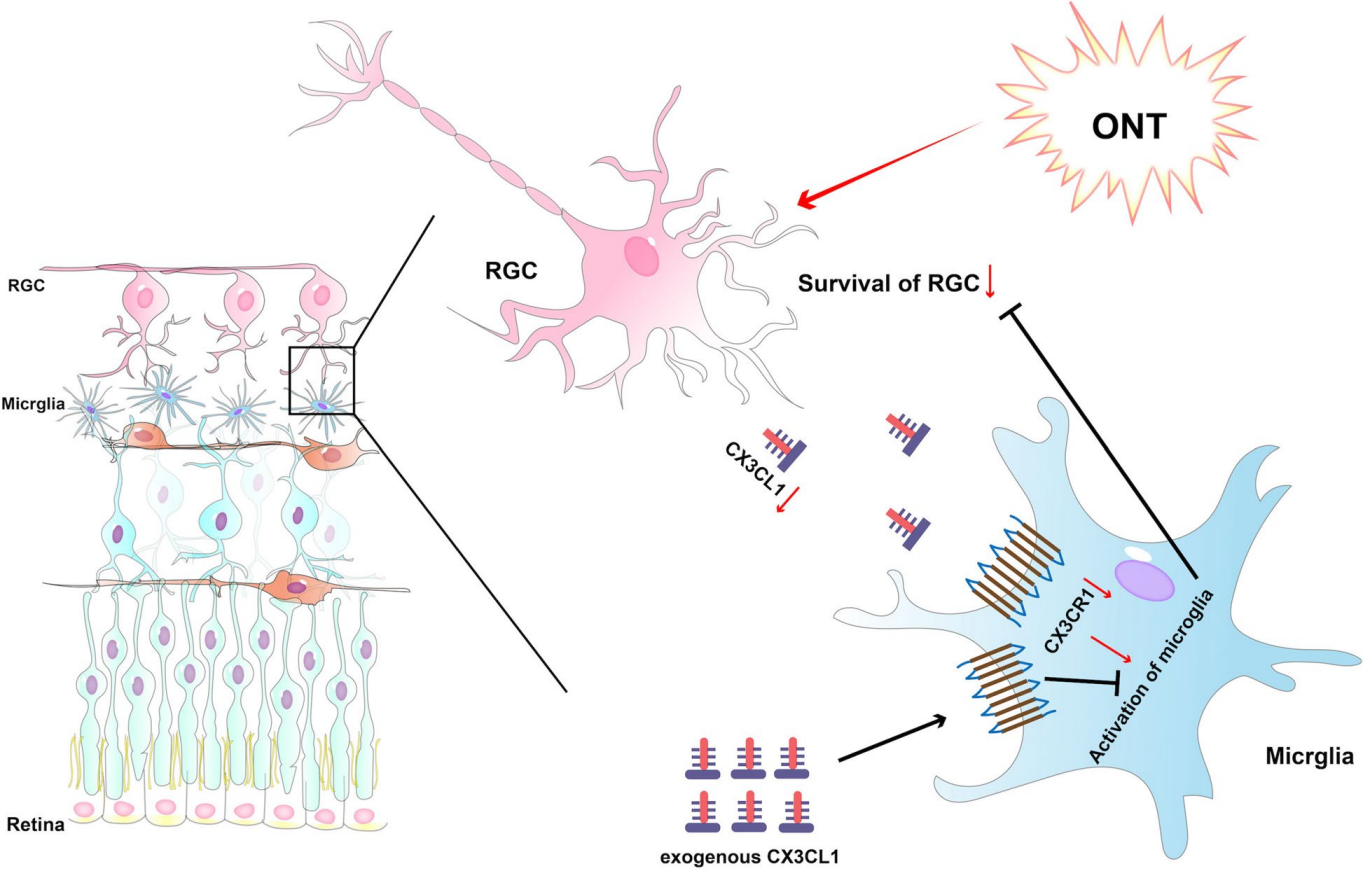
The crosstalk between retinal microglia and RGCs Via CX3CL1-CX3CR1 axis after ONT

The study explored the role of the CX3CL1-CX3CR1 axis in promoting the survival and axonal regeneration of RGCs by inhibiting microglial activation post-ONT (Fig. 7). The experimental results suggest a neuroprotective effect of 5 ng/mL CX3CL1 intravitreal injection administered to the ONT mouse model, the most optimal with delivery at 48 h post-ONT. This study provides a potential target for neuroprotective treatment of clinical optic nerve injuries. Future studies could further investigate the protective effect of the CX3CL1-CX3CR1 axis on optic nerve structure and function through controlled in vivo experiments.

## Data Availability

The datasets used and/or analyzed during the current study are available from the corresponding author on reasonable request.

## Abbreviations

ARVO: Association for Research in Vision and Ophthalmology
CD68: Cluster of Differentiation 68
CNS: Central nervous system
CSF-1R: Colony-stimulating factor 1 receptor
DMSO: Dimethyl sulfoxide
FKN: Fractalkine
F-VEP: Flash Visual evoked potentials
GAP-43: Growth-associated protein-43
Iba-1: Ionized calcium bindingadaptor molecule-1
ONT: Optic nerve trauma
PBS: Phosphate-buffered saline
RGCs: Retinal ganglion cells
SPF: Specific pathogen-free
